# Human exploitation assisting a threatened species? The case of muttonbirders and Buller’s albatross

**DOI:** 10.1371/journal.pone.0175458

**Published:** 2017-04-13

**Authors:** Susan M. Waugh, Timothée A. Poupart, Colin M. Miskelly, Jean-Claude Stahl, John P. Y. Arnould

**Affiliations:** 1 Museum of New Zealand Te Papa Tongarewa, Wellington, New Zealand; 2 Deakin University, 221 Burwood Highway, Burwood, Victoria, Australia; Hawaii Pacific University, UNITED STATES

## Abstract

Albatrosses are flexible and adaptable predators, relying on live prey as well as carrion. Use of predictable food sources and reliance on human-produced resources are well-known trait in long-range feeders like albatrosses and petrels. Breeding Buller’s albatrosses studied at Solander I. (Hautere), New Zealand fed their chicks the remains of sooty shearwater juveniles (*tītī* in Māori), which are harvested from nearby muttonbirding sites. Evidence of this food type was found at over 10% of nests examined, and 17–40% birds that were fitted with GPS loggers visited muttonbirding sites in this and previous studies. Muttonbirding is a traditional practice that has continued for centuries, with up to 120 tonnes of offal discharged to the sea annually during the present day harvest. It coincides with the energetically-demanding early chick period for the albatrosses. Our finding suggests that the offal may be an important, but overlooked element in the albatross diet. As an important supplementary food for the albatrosses it is likely to have contributed to the 3% per annum growth of their populations since the first comprehensive population surveys in 1969.

## Introduction

Many pelagic seabirds are opportunist feeders, relying on a mixture of predictable patches of natural prey [1, 2, 3] and as well as scavenging from the carcasses of dead vertebrates [4] fisheries waste [5] or offal from meat processing [6, 7]. Scavenged remains of marine mammals and birds makes up 5–51% of the diet of some albatross species [4].

Muttonbirding is a centuries-old practice [8, 9] undertaken by New Zealand Māori, and refers to the harvest of petrel chicks. Sooty shearwater (*Puffinus griseus*) chicks (*tītī* in Māori) are harvested in the mid-to-late fledging period [10]. Around Stewart I. / Rakiura (47°S, 167.84°E), the *tītī* harvest is undertaken by members of Ngai Tahu people [8] at the *tītī* islands [8, 11, 12] from 1 April to 31 May. Tribal elder, Rakiihia Tau, stated “Ngai Tahu's relationship with the *Tītī* Islands (*sic*) is undoubtedly a most important cultural, social and political facet of Ngai Tahu tribal identity” [8]. The *tītī* harvest, and traditional hunting grounds are recognised for their importance in cultural practice, maintaining social cohesion and traditional ecological knowledge, as well as providing an important source of income and food [8, 12, 13, 14]. The activity is regulated by statutory instruments [8, 14, 15] and traditional knowledge systems govern its sustainability [8, 9, 13]. Harvest levels reported from the early 20^th^ century suggested up to 250,000 chicks per year were harvested [16] with estimates of up 400,000 chicks in the 21^st^ century [17]. Archaeologists contend that large-scale muttonbirding operations commenced post 1800 [10], but there is a lack of evidence to confirm the scale of operations during the pre-European contact era.

Tracking studies of the globally threatened [18] Buller’s albatross *Thalassarche bulleri bulleri* were undertaken in this study to explore their present-day foraging behaviour. The species was studied extensively in the 1990s, with adult breeding birds satellite tracked from the two main breeding sites at Solander I (Hautere) (46.567°S, 166.883°E) and the Snares Is / Tini Heke (47.166°S, 166.53°E) [19, 20]. That work showed a concentration of foraging effort within 200 km of the breeding sites, particularly over continental shelf-areas, but birds also frequented more remote areas in the west, in the Tasman Sea and also to the east along the Chatham Rise. We measured fine-scaled movements of the albatrosses, with GPS locations recorded at 2 minute intervals, enabling an exploration of the movements of the birds, relative to geographical features. We also undertook incidental observations of food items rejected around nests, which included the remains of *tītī*. In this study, we explored the implications of albatrosses provisioning chicks from discards from muttonbirding practice and the possible benefits of this unusual feeding activity on the growth of the albatross population.

## Materials and methods

**Fig 1:**
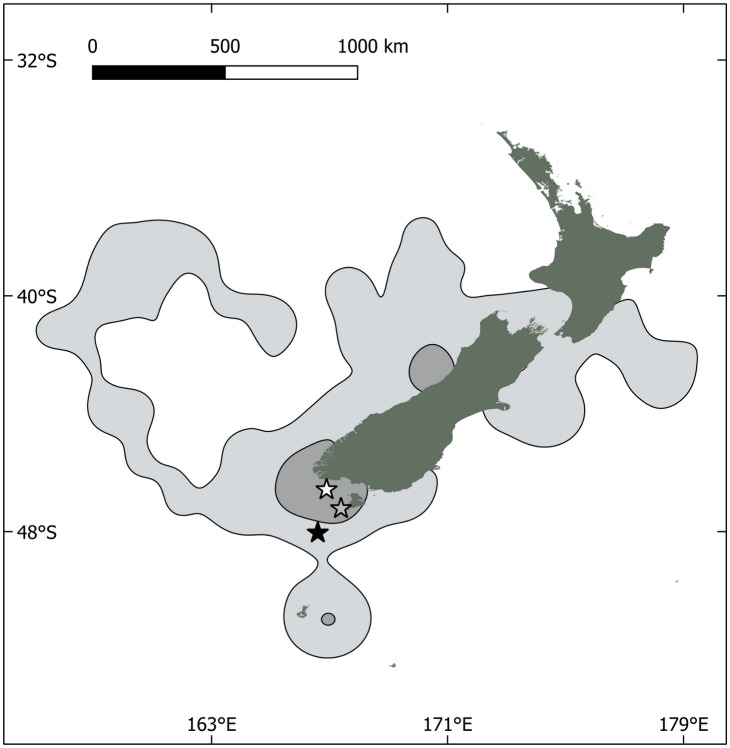
Foraging trips completed by 17 Buller’s albatross from Solander I. **(Hautere) (open star), showing 50% and 95% UDs**. Muttonbird Islands are shown with a grey star, and the second breeding site for the species at Snares Islands (Tini Heke) is shown with a black star.

**Fig 2:**
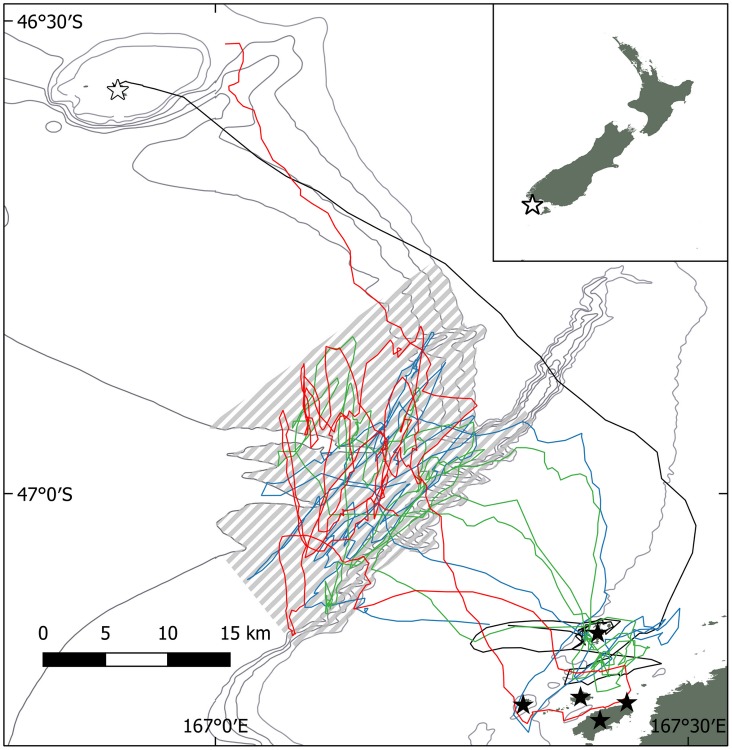
The foraging trip of a Buller’s albatross from Solander I. **(Hautere) (open star) for 4 d in May 2016 (day 1- black; 2-blue; 3-green; 4-red) of its 9.6 d trip, with a strong focus on the Muttonbird Is (black stars) west of Stewart I (Rakiura)**. This bird used the shelf-break area (hatched) on 8 d, and made 22 visits to muttonbirding sites. Dotted lines—bathymetric contours (50 m intervals). Dark grey–land areas.

The study involved non-invasive techniques applied to live birds from a protected species, and was assessed and approved by the Department of Conservation and Museum of New Zealand Te Papa Tongarewa research ethics groups. No adverse impacts of the study were detectable on either the study animals nor their progeny. Input was gathered from research colleagues with muttonbirding experience in the region visited by the seabirds studied. This was in accordance with Te Papa protocols for community consultation, and followed standard scientific practice for reporting personal communications. Approved animal handling procedures were granted, and entry to the publically owned but protected site were obtained prior to the study under permits 49827-FAU and 50212-LNZ issued by the Department of Conservation. Data relating to the foraging tracking data in Figs 1 and 2, and S1 Table on are available at **https://doi.org/10.6084/m9.figshare.4644751.v1**.

Buller’s albatross breeds only in New Zealand, with the population found at two breeding sites, 165 km apart–Solander I (Hautere) with a breeding population of approximately 4,900 pairs, and Snares Is / Tini Heke where an estimated 8,700 pairs bred, based on 2002 estimates [21]. At Solander I. (Hautere), 50 numbered Buller’s albatrosses nests were monitored from 10–27 May 2016. The research team of 4 was present on this remote, uninhabited island and visited study nests twice a day, but throughout daylight hours, all nests were continuously visually assessed from a distance of 5–50 m for visits by adults, presence of the chick and incidental observations, such as predation events. Regurgitated items at nests were noted at the study colony and adjacent areas. Twenty albatrosses with young at the nest were fitted with I-Got-U GPS loggers (GT-600 Mobile Action), repackaged in water-proof tubing and taped to back feathers using waterproof adhesive tape. The device deployed weighed 50g, or 1.5–2.5% of the adult birds weight (average, 2,592 g ± 300 g SD), and were programmed to record locations every 2 minutes. Seventeen loggers were retrieved. Data were groomed to remove locations at the island (e.g. for the minutes prior to and after deployment). Quantum GIS [22] was used to map the tracks in relation to bathymetry and surrounding land masses. Distances travelled, distances from one site to another, and times were calculated based on GIS software procedures, or custom software. Foraging locations from all birds were used to generate 50% and 95% utility distributions using the kernel-density estimation [23] with the *KernelUD* function in Program R [24], implementing using package ‘adehabitat’[25].

Interviews were conducted with three muttonbirders [26, 27, 28], two of whom were very experienced [26, 28]. The purpose of the study was explained, and participants were aware that their responses would be published as part of the research programme. We described the observations of bird regurgitates of shearwater remains and tracking locations, and asked these colleagues to assist us in interpreting our findings, to share their experiences and observations in relation to seabirds feeding on muttonbird remains, as well as details of the muttonbirding discarding practices at sites they were familiar with. Their responses were provided within this context, thereby according consent for their comments to be published. The interviews were intended to provide fine-detail to elaborate practices described in the literature, and in relation to the specific research findings of this study. Interviews were treated as ‘personal communications’ with research associates [26, 27, 28]. We sought input from colleagues who participate in muttonbirding at sites relevant to the study, and where harvest was occurring concurrent to the Te Papa bird tracking study.

## Results

### Shearwater remains fed to albatross chicks

Fresh *tītī* remains were found at five of the 50 study nests on 10–11 May 2016. These had been rejected by the chicks, in an area where native, but introduced weka *Gallirallus australis* could readily scavenge them, thus were likely to have been deposited 1–2 days beforehand. The items were: bolus of shearwater feathers (one nest); the head of a juvenile shearwater and feather bolus (one nest); feet and tarsi (three nests). At nests in two adjacent areas a feather bolus, feet and tarsi were found. Examples were retained in the Te Papa collections (TMP288136–288142).

### GPS-tracked albatrosses frequent muttonbirding sites

Seventeen albatross tracks were obtained and the combined set of tracks was used to produce kernels of usage of marine areas (Fig 1.). These kernels showed that most of the birds were feeding chicks using waters within Foveaux Strait, but several also fed at greater distances from the breeding site, some travelling to the Tasman Sea and north of Cook Strait. On average for the 40 foraging trips from the 2016 tracked birds travelled 1290 km (± 1853 km SD), during 2.45 d (± 3.2 d SD), with a range of 224 km (± 222 km SD).

One adult albatross was observed regurgitating *tītī* remains to its chick on 10 May. This bird was subsequently tracked, and spent time close the *tītī* islands near to Stewart I. / Rakiura during its 9.6 day trip (Fig 2). It visited five muttonbirding sites, spending 2–110 min within 2 km of the shore on 22 occasions (average 19 ± 28 min, S1 Table). It alternated these periods with visits to the shelf-break west of Stewart I. / Rakiura in bouts of 20–1,175 min (average 208 ± 264 min, n = 20). Visits to the muttonbirding sites were almost always between 06:45 h and 11:00 h. Two other tracked birds passed within 2 km of the muttonbirding sites on their return to Solander I. (Hautere) from feeding further to the east. These birds stayed 6 and 20 minutes at these sites, respectively.

### Muttonbirding practice at the tītī islands, Stewart I./Rakuria

*Tītī* are harvested from 1 April to 31 May annually. During the period when the albatross tracking study was undertaken, harvesting occurred at night-time [26, 27] as fully-feathered juvenile shearwaters emerge from their burrows at this time [11]. The *tītī* were caught, killed, and hung overnight, then plucked and preserved the following day [26, 27, 28]. The offal including heads, guts, and feathers was discarded in the sea adjacent to the islands from afternoon to evening [28].The exact timing varies between sites, in relation to the number of birds collected and workers processing them [11]. Scavenging seabirds were often seen using the offal [26, 27, 28]. One muttonbirder [26] noted that giant petrels (*Macronectes* sp.) came ashore frequently during the study period to feed from the offal, and that mollymawks (likely Buller’s albatrosses) congregated further offshore in the bay. Another [28] noted that although both giant petrels and Buller’s albatrosses were present in numbers in May 2016, the giant petrels dominated access to the offal, and kept the albatrosses at a distance. The muttonbirders noted that offal provides food to marine life at these sites [27, 28]. It is assumed that 35% by weight of the *tītī* is discarded [29], at an average weight of 700 g [16]– 860 g [29], this amounts to between 78 and 120 tonnes of offal annually. This proportion of the whole weight of chicks processed includes the bones, heads, feathers, viscera, feet, all of which were noted in our observations of offal being re-fed to chicks. Based on research into albatross diet [4], it is likely that all discarded parts of the *tītī* carcasses would be ingested by scavenging albatrosses.

## Discussion

Our observations indicated that Buller’s albatrosses are adaptable feeders, and in addition to feeding extensively on fisheries waste, as reported in the literature, there is evidence that around 10% or more adults and chicks fed on *tītī* remains from our direct observations. These are likely to have originated from the offal of the traditional Māori harvest (see below). The offal is discarded to the sea, and forms a predictable resource of an estimated 78–120 tonnes per annum for marine species, in an area that is central to the Buller’s albatross foraging range during the early chick period (Fig 1). *Tītī* remains were found at 10% of monitored albatross nests at Solander I. (Hautere) over the initial two-day period of a 2-week long observation period. These observations concerned only the indigestible remains of *tītī*, and it is likely that a greater proportion of chicks were fed soft body parts of *tītī* over the two-month harvest period in April and May, and a higher proportion of foraging adults visited these sites over the season.

### Is muttonbirding offal the likely source of the observed tītī remains?

We examined the evidence that the *tītī* remains observed at the albatross colony originated from offal, rather than from natural mortality or depredation of juvenile shearwaters following fledging. Wrecks of shearwaters have been reported around New Zealand during their fledging period in May, October and November over several decades [30], and during May 2016, dead and dying shearwater juveniles were observed along southern Stewart Island / Rakiura coastal areas (N. Cobb, pers. comm). While we cannot rule out the possibility that some of the remains observed at the albatross colony originated from the natural mortality of shearwater juveniles at sea, it is unlikely that albatrosses would access these remains on beaches, where many specimens accumulate, as albatrosses are not known to feed on land [31]. In contrast, giant petrels are likely to depredate or scavenge dead and dying birds on the shore, and were observed feeding on *tītī* offal at the shore line at one muttonbirding site during the study [28]. Had the albatrosses fed on floating moribund *tītī* at sea, we would expect them to have eaten soft body parts such as muscle and viscera, and not to ingest heads, feathers, tarsi and feet. This is because these elements are firmly attached to a whole carcass, and the whole carcass is too large for these small albatrosses to swallow. It, therefore, seems more likely that the observed *tītī* remains came from offal which is discarded as discrete body parts into the sea, rather than from the beach wrecked or floating carcasses of juvenile shearwaters.

### Albatrosses change their foraging zone when offal is available

We explored whether there were changes in the albatrosses foraging behaviour between periods with and without harvest, reported in the literature. Tracking studies of breeding Buller’s albatross from the 1990s [19, 20] showed that while 5–20% of tracked birds fed near to the *tītī* islands during incubation, the proportion increased to 40% during the first few weeks of chick-rearing. This change in feeding activity is likely to represent a combination of a) the need to feed chicks often early in the brood period, with adults reducing their foraging ranges at this time, and b) the opportunity to exploit offal from muttonbirding practice, which occurs in the central part of the range of this albatross species, within 120 km of the nesting sites for both populations.

### The timing of albatross hatching coincides with the provision of offal

The mean hatch date for Buller’s albatross on 2 April [32]occurs at the same time as the *tītī* harvest starts each year, 1 April. The provision of offal increases through the first month of the albatross chick-rearing as the *rama* hunting period, when most *tītī* are harvested, occurs from 25 April– 30 May [33]. Thus the timing and availability of offal is likely to be a food resource upon which albatrosses from nearby colonies can rely when raising young chicks [34], recognised as the most constraining stage in the 5-month long chick-rearing period for albatrosses.

### Offal feeding albatross spent its foraging trip visiting muttonbirding sites

In addition to observing *tītī* remains at the albatross colony, one of 17 Buller’s albatrosses tracked in May 2016 made 22 searches near *tītī* harvest sites during a period that offal from muttonbirding was available at these particular sites, with active muttonbirding activity at these islands in 2016 (Fig 2). This bird fed a *tītī* head to its chick, along with *tītī* feathers. Two other GPS-tracked birds flew close to active muttonbirding sites during the study, but stayed for short periods only (less than 20 mins each). The behaviour of albatrosses and other carrion feeding birds at the muttonbirding sites observed by workers on the harvest corroborates the information from the GPS tracking, that Buller’s albatross frequented the sites, but remained at some distance from the shore [28].

### Buller’s albatross diet

The use of *tītī* remains as a food by Buller’s albatross has not been noted previously, although 3% of diet samples analysed in the 1990s contained bird feathers [35]. However, these authors [35] noted that “bird feathers of non-penguin origin were present in eight May samples, sometimes in large numbers, and suggested scavenging on carcasses”. The timing in the season of these observations in the 1990s coincides with the availability of carcasses and offal from the *tītī* harvest.

Albatrosses and petrels are known as adaptable scavengers, feeding from a variety of natural and anthropogenic food sources, as well as capturing live prey [4]. For example, in past decades, they were frequently seen around meat-works effluent outfalls [7, 36, 37] until improvements in waste-treatment practices eliminated this food source from the 1960 to 1980s [7]. It is therefore not surprising that they would make use of the offal from *tītī* harvest, but our observations highlight the inter-dependence of marine species and traditional practices in a hitherto undocumented manner.

Offal from muttonbirding is comprised of the viscera, wings, tails, heads, feathers, tarsi and feet of the *tītī* [29]. These elements of the carcass can provide important nutrients and minerals if digested. Buller’s albatross are known to ingest fish guts and frames from fishing vessels, as well as feeding on more natural prey such as fish and squid, and invertebrates such as crustacean and salps [35]. Although offal may seem to be of little nutritive value, it contains the building blocks of seabird muscle, bone and feather, and is presented in a readily digestible parcels, with dissected body parts that could be easily swallowed whole by an albatross. It, therefore, seems likely that an albatross would readily eat offal of this type, and derive benefit from it.

### Population implications of albatross feeding on tītī offal

The provision of *tītī* offal for Buller’s albatrosses may be contributing to the overall population growth of the species’ two breeding populations, which are subject to a number of threats to their population viability, such as mortality in trawl and longline fisheries [38, 39]. The food supplied by muttonbirding offal may have benefited the two populations of Buller’s albatross over time, as it is within easy reach (less than 120 km from the two breeding populations) during the short trips required to feed chicks immediately after hatching. The population of albatrosses has increased at around 3% p.a. since surveys began in 1969. Although undocumented, the populations may have been recovering from human exploitation during the sealing era in the 1800s at both of the breeding sites. Sealers were stationed or marooned at both breeding sites over several years [40], and it is highly likely that they harvested the albatrosses for food during this period. Thus, the *tītī* harvest may have assisted in the population rebuild over many decades prior to scientific surveys of the populations. Buller’s albatrosses feed chicks substantially on trawl discards (42% - 60% by weight of diet samples) [35]. While fisheries waste has no-doubt contributed to population increases, recent modelling studies showed that the negative populations effects of occasional fisheries mortality has not affected the viability of the populations [39]. Feeding on *tītī* offal is another important factor to consider in the evolution of the albatross populations. This food source varies with El Niño Southern Oscillation [41], a fact which may amplify any climate-related changes in chick survival for the albatrosses.

The intensity of muttonbird harvest is reducing over time as the community of muttonbirders changes [9, 11], and will also vary with climatic conditions and long-term declines in shearwater populations [42]. As a result, the food provided to albatrosses and other marine scavengers from *tītī* offal may decrease. Our findings show that while muttonbirding is recognised for its value in maintaining cultural traditions, it may also be important in sustaining this threatened species.

## Supporting information

S1 Table Times spent by a three adult breeding Buller’s albatross during chick-rearing stage. Bouts of time in proximity muttonbirding sites (shaded) and within the shelf-break area (unshaded) during a 9.6 d trip on 11–19 May 2016 for bird M83587, a 4 day trip by bird M84344 (data from Day 4 only shown) and a 10 day trip for Bird M48065 (data from Day 10 only shown). Days are separated by horizontal lines in the table.
(DOCX)
