# Supplementary material for: Human exploitation assisting a threatened species? The case of muttonbirders and Buller’s albatross
Source: PLoS One. 2017 Apr 13;12(4):e0175458. doi: 10.1371/journal.pone.0175458 (PMC5391021; doi:10.1371/journal.pone.0175458)
Supplement: S1 Table — Bouts of time in proximity muttonbirding sites (shaded) and within the shelf-break area (unshaded) during a 9.6 d trip on 11–19 May 2016 for bird M83587, a 4 day trip by bird M84344 (data from Day 4 only shown) and a 10 day trip for Bird M48065 (data from Day 10 only shown). Days are separated by horizontal lines in the table. (DOCX) [file pone.0175458.s001.docx]

S1 Table.

| Bird ID | Day of trip | Site by Day | Start of first 2 min interval | Start of last 2m interval | Duration (min) |
| --- | --- | --- | --- | --- | --- |
| M83587 | 1 | Putauhinu I | 12/05/16 08:58 | 12/05/16 09:04 | 4 |
|  |  | Mokinui I (Big Moggy I) | 12/05/16 10:00 | 12/05/16 11:50 | 110 |
|  |  | Shelf-break | 12/05/16 12:49 | 13/05/16 05:17 | 988 |
| M83587 | 2 | Pohowaitai I | 13/05/16 06:49 | 13/05/16 06:59 | 10 |
|  |  | Putauhinu I | 13/05/16 07:06 | 13/05/16 07:09 | 3 |
|  |  | Mokinui I (Big Moggy I) | 13/05/16 08:22 | 13/05/16 09:47 | 85 |
|  |  | Shelf-break | 13/05/16 10:32 | 13/05/16 10:52 | 20 |
|  |  | Putauhinu I | 13/05/16 10:55 | 13/05/16 10:57 | 2 |
|  |  | Shelf-break | 13/05/16 10:58 | 13/05/16 16:00 | 302 |
|  |  | Mokinui I (Big Moggy I) | 13/05/16 16:17 | 13/05/16 16:40 | 23 |
|  |  | Putauhinu I | 13/05/16 16:42 | 13/05/16 16:45 | 3 |
|  |  | Mokinui I (Big Moggy I) | 13/05/16 16:46 | 13/05/16 16:54 | 8 |
|  |  | Shelf-break | 13/05/16 17:55 | 14/05/16 03:39 | 587 |
| M83587 | 3 | Mokinui I (Big Moggy I) | 14/05/16 07:24 | 14/05/16 07:26 | 2 |
|  |  | Putauhinu I | 14/05/16 09:13 | 14/05/16 09:16 | 3 |
|  |  | Mokinui I (Big Moggy I) | 14/05/16 10:01 | 14/05/16 10:43 | 42 |
|  |  | Shelf-break | 14/05/16 11:43 | 15/05/16 07:18 | 1175 |
| M83587 | 4 | Rerewhakaupoko I (Solomon I) | 15/05/16 07:54 | 15/05/16 08:16 | 22 |
|  |  | Taukihepa (Big South Cape I) | 15/05/16 08:16 | 15/05/16 08:24 | 8 |
|  |  | Putauhinu I | 15/05/16 08:25 | 15/05/16 08:30 | 5 |
|  |  | Pohowaitai I | 15/05/16 08:42 | 15/05/15 09:02 | 21 |
|  |  |  | 15/05/16 09:48 | 15/05/16 10:48 | 60 |
|  |  | Shelf-break | 15/05/16 18:29 | 15/05/16 21:27 | 178 |
|  |  |  | 15/05/16 21:51 | 16/05/16 00:32 | 161 |
| M83587 | 4 | Shelf-break | 16/05/16 01:34 | 16/05/16 07:26 | 352 |
|  |  | Taukihepa (Big South Cape I) | 16/05/16 08:31 | 16/05/16 08:34 | 3 |
|  |  | Putauhinu I | 16/05/16 08:35 | 16/05/16 09:25 | 50 |
|  |  | Rerewhakaupoko I (Solomon I) | 16/05/16 08:25 | 16/05/16 08:31 | 6 |
|  |  | Pohowaitai I | 16/05/16 09:31 | 16/05/16 09:34 | 3 |
|  |  | Shelf-break | 16/05/16 10:14 | 16/05/16 10:50 | 36 |
|  |  |  | 16/05/16 11:35 | 16/05/16 15:29 | 234 |
| M83587 | 5 | Shelf-break | 17/05/16 17:15 | 17/05/16 19:57 | 162 |
|  |  |  | 17/05/16 21:17 | 17/05/16 22:35 | 78 |
| M83587 | 6 | Shelf-break | 18/05/16 00:10 | 18/05/16 00:56 | 46 |
|  |  | Pohowaitai | 18/05/16 07:44 | 18/05/16 07:48 | 4 |
|  |  | Mokinui I (Big Moggy I) | 18/05/16 08:05 | 18/05/16 08:16 | 11 |
|  |  |  | 18/05/16 12:56 | 18/05/16 14:15 | 79 |
|  |  | Shelf-break | 18/05/16 18:35 | 18/05/16 19:30 | 55 |
|  |  |  | 18/05/16 21:26 | 18/05/16 22:58 | 92 |
|  |  |  | 18/05/16 23:31 | 19/05/16 03:15 | 224 |
| M83587 | 7 | Shelf-break | 19/05/16 04:21 | 19/05/16 07:08 | 167 |
|  |  |  | 19/05/16 08:19 | 19/05/16 09:04 | 45 |
| M84344 | 4 | Poutama I | 14/05/2016 09:15 | 14/05/2016 09:28 | 13 |
|  |  | Pohowaitai | 14/05/2016 09:47 | 14/05/2016 09:53 | 6 |
| M48065 | 10 | Poutama I | 19/05/2016 21:07 | 19/05/2016 21:11 | 6 |
